# Baseline characteristics of European and non-European adult patients with attention deficit hyperactivity disorder participating in a placebo-controlled, randomized treatment study with atomoxetine

**DOI:** 10.1186/1753-2000-7-14

**Published:** 2013-05-06

**Authors:** Himanshu Upadhyaya, Lenard A Adler, Miguel Casas, Alexandra Kutzelnigg, David Williams, Yoko Tanaka, Jody Arsenault, Rodrigo Escobar, Albert J Allen

**Affiliations:** 1 Lilly Research Laboratories, Lilly Corporate Center, Indianapolis, IN 46285, USA; 2 New York University School of Medicine, New York University Medical Center and NY VA Harbor Healthcare Service, 650 First Ave., 7th Floor, New York, NY, 10016, USA; 3 Servicio de Psiquiatria, Hospital Universitari Vall d'Hebron, Universidad Autonoma de Barcelona, Passeig Vall d'Hebron, 119-129, Barcelona, 08035, Spain; 4 Universitätsklinik für Psychiatrie und Psychotherapie, Abteilung für Biologische Psychiatrie, Medizinische Universität Wien, Währinger Gürtel 18-20, Wien, 1090, Austria; 5 PharmaNet/i3, an inVentiv Health Company, Indianapolis, IN, 46280, USA; 6 Lilly Research Laboratories, Sannomiya Plaza Bldg. 7-1-5, Isogami Dori, Chuo-ku, Kobe, 651-0086, Japan

**Keywords:** Attention deficit/hyperactivity disorder, Adult, European, Atomoxetine

## Abstract

**Background:**

Attention deficit/hyperactivity disorder (ADHD) often presents as an impairing lifelong condition in adults; yet it is currently underdiagnosed and undertreated in many European countries. This analysis examines the characteristics of adult patients with ADHD in a European (EUR) and non-European (NE) patient population.

**Methods:**

Baseline data from the open-label treatment period of a randomized trial of atomoxetine in adult patients with ADHD (*N*=2017; EUR, *n*=1217; NE, *n*=800) were examined. All patients who were enrolled were included in the baseline analyses.

**Results:**

The demographics for patients in the EUR and NE groups were comparable. Patients in the EUR group had a somewhat lower percentage of prior exposure to psychostimulants compared with the NE group (32.7% vs. 38.9%, *p*=.0049). Scores on the Conners’ Adult ADHD Rating Scale-Investigator Rated: Screening Version with adult ADHD prompts (18-item total, inattentive and hyperactive/impulsive subscales, and index) were comparable. The adult ADHD Quality of Life-Life Outlook and Life Productivity domain scores were significantly different between groups (*p*≤.0004). The EuroQol-5 Dimension United Kingdom and United States population-based index scores and Health State score were comparable between groups.

**Conclusions:**

Adults with ADHD in Europe present similar demographics and baseline characteristics to those outside Europe and hence, study results outside Europe may be generalizable to patients in Europe.

**Trial registration:**

Clinicaltrials.gov, NCT00700427

## Background

Attention deficit/hyperactivity disorder (ADHD) is a neuropsychiatric disorder that begins in childhood. This disorder persists into adulthood in around one-third to two-thirds of children with ADHD [1-9]. Epidemiological studies have estimated the worldwide prevalence of ADHD in adults to range from 2% to 7% [10-14].

Attention deficit/hyperactivity disorder remains a poorly understood disorder in adults and often presents as an impairing lifelong disorder currently underdiagnosed and undertreated in many European (EUR) countries [10,15,16]. In many EUR countries, professionals working in the adult mental health field may not know that ADHD frequently persists into adulthood, how ADHD presents in adults, and the impairment ADHD causes in adults [16]. Due to the lack of understanding of adult ADHD diagnosis and treatment, many adults with ADHD are misdiagnosed and do not receive effective treatments.

One reason for underdiagnosis and undertreatment of ADHD in adults is that ADHD symptoms differ throughout the patient’s lifespan. Adult patients experience more subtle symptoms such as impairments in executive function (time management, organization, and goal-oriented activity), difficulties in relationships, poor motor vehicle driving skills, and psychiatric comorbidities including substance abuse disorders [12,17,18]. Additionally, compared with the current situation, many adults presenting as ADHD patients today were not diagnosed during their childhoods since the diagnosis and treatment of ADHD was less accepted in child psychiatric practice in Europe during the 1980s and 1990s [19]. The stigma that surrounds ADHD and the presence of comorbid psychiatric disorders also contributes to the underdiagnosis of ADHD in adults. Undertreatment is a consequence of underdiagnosing the condition in adults and can occur due to a lack of understanding of available treatments, particularly stimulant medication treatment. This can lead to reluctance by the patient to seek treatment and reluctance by medical professionals to diagnose and treat ADHD in adults [20].

Taking the differences in diagnosis and treatment of adult ADHD in many EUR regions into account, additional information on the characteristics of adult ADHD patients within Europe is needed [16,19,21]. The characteristics of ADHD relate in part to cultural expectations, which may explain why diagnostic and treatment rates of ADHD are higher in the United States (US) than in many EUR countries. The full extent to which ADHD characteristics vary across countries has not been systematically studied. While there are many similarities between children with ADHD across countries, it is suspected that the differing cultures, traditions, and child and adolescent mental health services across countries will result in large variations in the rate of ADHD diagnosis and in clinical practice.

The purpose of this analysis was to examine the characteristics of adult patients with ADHD in a large patient population in Europe as well as those outside Europe.

## Method

The subjects in this analysis included adults with ADHD who participated in a multicenter, multinational, randomized withdrawal trial of atomoxetine with 3 study periods: screening/washout, open-label acute treatment, and a randomized, double-blind, placebo-controlled withdrawal. Study Period I was a screening/washout period that consisted of a minimum of 3 days and a maximum of 28 days. Study Period II was a 12-week open-label treatment period (*N*=2017; EUR, *n*=1217 [60%]; non-European [NE], *n*=800 [40%]). The majority (60%) of patients in the study were from Europe; of the remaining patients, the overwhelming majority (75%) were from the US. The numbers from other countries were relatively smaller and hence, we combined them with the US as non-European. Additionally, we carefully considered in which group to include Russia, which is considered part of Europe but is also part of Asia. However, since Russia is not part of the European Union, we chose not to include Russia in the EUR group. In addition, there were only 6 patients from Russia, so most likely the influence of Russian patients, overall, was limited. The NE group consisted of patients in the US (*n*=602), Canada (*n*=60), Mexico (*n*=53), Puerto Rico (*n*=52), Argentina (*n*=27) and Russia (*n*=6). The EUR group consisted of patients in Germany (*n*=434), Spain (*n*=153), Belgium (*n*=137), Sweden (*n*=112), Austria (*n*=109), Netherlands (*n*=71), France (*n*=65), Finland (*n*=52), Italy (*n*=32), Denmark (*n*=15), United Kingdom (UK) (*n*=27), Switzerland (*n*=7), and Portugal (*n*=3). Any excluded medications were tapered off and/or stopped during this study period.

An informed consent document (ICD) approved by an Ethical Review Board or similar body was signed by the patient and/or representative deemed appropriate, according to local laws and regulations. Informed consent was obtained before any changes were made to the patient’s medical treatment plan for the purpose of study participation and before any study procedures were performed. The study was conducted in accordance with the Declaration of Helsinki and the applicable laws and regulations of the study countries and regions. The study is currently ongoing; the primary outcome of this trial will be reported upon study completion.

Eligible patients were adults (aged 18 to 50, inclusive at the time of signing the ICD) with ADHD, as established by the Conners’ Adult ADHD Diagnostic Interview for Diagnostic and Statistical Manual of Mental Disorders Fourth Edition (DSM-IV) (CAADID), who had ADHD symptoms in childhood and scored ≥2 on at least 6 items of either the inattentive and/or hyperactive/impulsive core subscales of the Conners’ Adult ADHD Rating Scale (CAARS)-Investigator Rated: Screening Version (CAARS-Inv:SV). CAARS-Inv:SV is a 30-item scale containing 2 subscales (inattentive and hyperactive/impulsive) and the ADHD index [22]. The inattentive and hyperactive/impulsive subscales each include 9 items, and the ADHD index includes 12 items [22]. Each item is scored from 0 to 3 (0=not at all, never; 3=very much, very frequently), and an ADHD symptom is considered to be present if the score on the corresponding item is ≥2. The CAARS-Inv:SV was administered with adult ADHD prompts [23]. In addition, the CAARS-Inv:SV 18-item total ADHD symptom score had to be ≥20, and patients had to have a score of ≥2 on at least 6 items of either the inattentive and/or hyperactive/impulsive core subscales of the CAARS-Observer Rated: Screening Version (CAARS-O:SV) for current symptoms.

The CGI-ADHD-S is a single-item clinician rating of the clinician’s assessment of the overall severity of the patient’s ADHD in relation to the clinician’s total experience with ADHD patients. Severity is rated on a 7-point scale (1=normal, not at all ill; 7=among the most extremely ill patients) [24]. Clinical Global Impressions-ADHD-Severity (CGI-ADHD-S) scores at screening had to be ≥4, which indicates at least moderate symptom severity.

All raters were trained in administration of the CAADID and CAARS-Inv:SV with adult prompts via standard training procedures [25]. Before starting the study, efficacy raters were required to attend a training session in which observed interviews and group discussion were used to standardize rating practices for the CAARS-Inv:SV. The process involved the following: 1) review of adult ADHD, 2) review of ADHD rating instruments, along with rating conventions, 3) presentation of a patient interview, rating the primary outcome measure (the total ADHD symptom score on the CAARS-Inv:SV with adult ADHD prompts), and discussion of “gold-standard” ratings (training rating), and 4) certification rating presentation of another patient interview of the total ADHD symptom score on the CAARS-Inv:SV with adult ADHD prompts. In order to be certified, raters had to agree (>80%) with the gold-standard total ADHD symptom score on the CAARS-Inv:SV with adult ADHD prompts on the certification rating.

Exclusion criteria stated that patients should be excluded from the study if they met the following criteria: Patients met full DSM-IV-Text Revision™ (DSM-IV-TR™) diagnostic criteria for any history of bipolar disorder, current major depression, a current anxiety disorder (including generalized anxiety disorder, panic disorder, or social phobia), or any history of a psychotic disorder. Additional exclusion criteria were as follows: Patients were currently using alcohol, drugs of abuse, or any prescribed or over-the-counter medication in a manner that the investigator considered indicative of chronic abuse or met DSM-IV-TR criteria for alcohol or other substance dependence.

### Statistical analyses

All patients who were enrolled in the open-label acute treatment period were included in this analysis of ADHD characteristics. Data at baseline were used in the current analysis.

Continuous baseline patient characteristics were summarized by mean, median, standard deviation, minimum, and maximum. Categorical baseline characteristics were summarized as percentages. Statistical comparisons were conducted using t-tests for continuous data and Fisher’s exact test or a chi-square test for categorical data; however, due to a large sample size (*N*=2017), some comparisons were too sensitive and detected unsubstantial differences. Hence, *p*-values were used as references only and standardized differences were used to determine clinical relevance as described below. Data on education level difference and family history were analyzed post hoc. The overall relationship of education level to prior stimulant use was assessed with a chi-square test, while Fisher’s exact test was used to test prior stimulant use for each individual level of education. The relationship of adult ADHD Quality of Life (AAQoL) scores to education level was assessed with analysis of variance (ANOVA). All tests were 2-sided and employed a significance level of 0.05. Statistical analyses were performed using SAS version 9.1.

In order to determine clinically relevant significance, the standardized differences were calculated for the CAARS-Inv:SV, CGI-ADHD-S, EuroQol-5 dimension (EQ-5D) Index, and AAQoL scores. The standardized differences between groups were calculated with the following formula: ([the mean for the EUR group] minus [the mean for the NE group]) divided by the pooled (or overall) standard deviation, thus mirroring the calculation of Cohen’s d [26]. The standardized difference is a measure of the relative size of the means and can be interpreted similar to Cohen’s d. Clinically meaningful differences were estimated with an anchor-based (one-half standard deviation) approach based on published literature [27,28]. Hence, standardized differences of 0.5 or higher were considered clinically meaningful. Although one-half standard deviation was developed as an anchor for health-related quality of life, for CAARS-Inv:SV and CGI-ADHD-S standardized differences, the same one-half standard deviation was used as a reference.

## Results

Baseline demographics were first compared among North America (N=767), South America (N=27) and Russia (N=6) to ensure homogeneity within NE, and ensure that differences between NE regions did not skew data. Age, gender, and ADHD subtype were examined but were not statistically different across the sub-regions (North America, South America, and Russia). The CAARS-Inv:SV total score, CGI-ADHD-S score, and AAQoL total score were compared among regions. All 3 CAARS scores (total, inattention, hyperactivity-impulsivity) were examined and all were significant (*p*<.0008). The CAARS-Inv:SV total score was significantly different (*p*<.0001), and it seemed to be due to the lower scores in Russia (mean=27.3) compared with North America (mean=39.6) and South America (mean=42.0). The CGI-ADHD-S analysis was not significantly different across regions (*p*=.0761) and neither was the AAQoL total score (*p*=.2662). Only CAARS-Inv:SV total score showed any potentially meaningful differences among North America, South America, and Russia, while all of the others did not; also, Russia’s sample was thought to be too small (N=6) to skew the NE data as a whole, justifying the grouping of these regions. In addition, Russia's sample (N=6) was too small to be a stand-alone region.

The demographics for patients in the EUR region and NE regions were compared (Table 1). The mean age of patients was 33.0 years in the EUR region and 33.4 years in the NE regions. The percentage of patients under 25 was 24.5% for EUR patients and 20.0% for NE patients. As expected, the breakdown for race was different in the comparison between NE and EUR groups, with the NE group having a higher percentage of Hispanic (18.8% vs. 0.7%) and African patients (6.4% vs. 0.4%). The gender distribution was approximately 59% male and 41% female for both patient groups. Mean weight was higher in the NE group (82.4 kg) compared with the EUR group (76.8 kg, *p*<.0001). Mean height was 173.8 cm for EUR patients and 171.7 cm for NE patients (*p*<.0001). Patients in the EUR group had a lower percentage of exposure to psychostimulants (32.7%) compared with patients in the NE group (38.9%, *p*=.0049). The ADHD combined subtype comprised 71.7% of the EUR group and 77.9% of the NE group, while the inattentive subtype comprised 24.5% of the EUR group and 21.0% of the NE group (*p*=.0001). Family history of ADHD in the patient’s mother (EUR = 5.2%, NE = 5.8%, not significant) or father (EUR = 8.5%, NE = 7.9%, not significant) was reported to be of similar frequency in the EUR and NE groups. Family history of ADHD in siblings was greater in the NE group (18.3%) compared with the EUR group (12.8%, *p*=.0043). Family history of ADHD in children was greater in the EUR group (25.3%) compared with the NE group (19.9%, *p*=.0343).

**Table 1:** Demographics

**Variable**	**EUR group ( *N *=1217)**	**NE group ( *N *=800)**	**Total ( *N *=2017)**	***p*-value**
Age in Years (Mean ± *SD*)	33.0 ± 9.2	33.4 ± 8.9	33.2 ± 9.1	.3999^b^
Age <25, *n* (%)	298 (24.5)	160 (20.0)	458 (22.7)	.0195^a^
Age ≥25, *n* (%)	919 (75.5)	640 (80.0)	1559 (77.3)	
Race, *n* (%)				<.0001^a^
Caucasian	1192 (97.9)	573 (71.6)	1765 (87.5)	
African	5 (0.4)	51 (6.4)	56 (2.8)	
Hispanic	9 (0.7)	150 (18.8)	159 (7.9)	
Other	11 (0.9)	24 (3.0)	35 (1.7)	
Gender, *n* (%)				
Male	702 (57.7)	482 (60.3)	1184 (58.7)	.2673^a^
Female	515 (42.3)	318 (39.8)	833 (41.3)	
Body Weight (kg), Mean (*SD*)	76.8 (17.6)	82.4 (19.7)	79.0 (18.7)	<.0001^b^
Height (cm), Mean (*SD*)	173.8 (9.4)	171.7 (10.0)	173.0 (9.7)	<.0001^b^
Prior Exposure to Psychostimulants, *n *(%)				
Yes	398 (32.7)	311 (38.9)	709 (35.2)	.0049^a^
No	819 (67.3)	489 (61.1)	1308 (64.8)	
ADHD Subtype, *n *(%)				.0001^a^
Inattentive	298 (24.5)	168 (21.0)	466 (23.1)	
Hyperactive/Impulsive	40 (3.3)	7 (0.9)	47 (2.3)	
Combined	872 (71.7)	623 (77.9)	1495 (74.1)	
Family History of ADHD, *n *(%)				
Children	308 (25.3)	159 (19.9)	467 (23.2)	.0343^a^
Mother	63 (5.2)	46 (5.8)	109 (5.4)	.2753^a^
Father	104 (8.5)	63 (7.9)	167 (8.3)	.2532^a^
Siblings	156 (12.8)	146 (18.3)	302 (15.0)	.0043^a^

Abbreviations: ADHD = attention-deficit/hyperactivity disorder; EUR = European; *N* = total number of patients; *n *= number of patients in the specified category; NE = non-European; SD = standard deviation.

^a^*p*-values are from Fisher’s exact test.

^b^*p*-values are from *t*-test.

The CAARS-Inv:SV ADHD index score was statistically significantly different between groups, as were the investigator-rated Hyperactivity-Impulsivity and Inattention subscale scores, and total ADHD symptom score (Table 2). The scores had differences between groups that ranged from 0.5 to 1.9 points. The CAARS-Inv:SV subscale scores were not clinically meaningfully different when evaluated with standardized differences (standardized differences = -0.191 to 0.38). The CGI-ADHD-S score was significantly lower in the NE group (4.7) compared with the EUR group (5.2) (*p*<.0001). The CGI-ADHD-S score at baseline was severe (mean=5.0 for EUR and NE combined, 4 is moderately ill and 5 is markedly ill), which suggests that the patients had significant impairment. When evaluated with standardized differences, the CGI-ADHD-S score was moderately different (EUR mean = 5.2, NE mean = 4.7, standardized difference = 0.625).

**Table 2:** ADHD symptom severity

**Variable mean ( *SD *)**	**EUR group ( *N *=1217)**	**NE group ( *N *=800)**	**Total ( *N *=2017)**	***p*-value**^**a**^	**Standardized differences**
CAARS-Inv:SV Score					
ADHD Index	24.7 (4.8)	22.8 (5.1)	23.9 (5.0)	<.0001	0.38
Hyperactivity-Impulsivity Subscale^b^	17.4 (5.2)	18.1 (5.0)	17.7 (5.1)	.0029	-0.137
Inattention Subscale^b^	20.9 (3.6)	21.4 (3.7)	21.1 (3.7)	.0008	-0.135
Total ADHD Symptom^b^	38.3 (6.7)	39.6 (7.0)	38.8 (6.8)	<.0001	-0.191
CGI-ADHD-S Score	5.2 (0.8)	4.7 (0.7)	5.0 (0.8)	<.0001	0.625
AAQoL					
Abbreviated Total Score^b^	47.6 (14.3)	48.4 (14.1)	47.9 (14.2)	.2035	-0.056
Psychological Health Section Score	51.4 (20.9)	52.5 (19.8)	51.9 (20.5)	.2379	-0.054
Relationships Section Score	53.6 (21.2)	53.7 (19.2)	53.6 (20.4)	.9101	-0.005
Life Outlook Section Score	45.1 (17.0)	52.5 (15.5)	48.1 (16.8)	<.0001	-0.440
Life Productivity Section Score	44.2 (18.1)	41.2 (19.1)	43.0 (18.6)	.0004	0.161
EQ-5D					
UK Population-Based Index Score	0.80 (0.21)	0.84 (0.18)	0.82 (0.20)	.0003	-0.200
US Population-Based Index Score	0.84 (0.14)	0.86 (0.13)	0.85 (0.14)	.0001	-0.143
Health State Score	70.0 (21.7)	75.6 (22.3)	72.2 (22.1)	<.0001	-0.253

Abbreviations: ADHD = attention-deficit/hyperactivity disorder; AAQoL = adult ADHD quality of life; CAARS-Inv:SV = Conner’s adult ADHD rating scale-investigator rated: Screening Version; CGI-ADHD-S = clinical global impressions–ADHD-severity; EQ-5D = EuroQol-5 dimensions; EUR = European; *N *= total number of patients; NE = non-European; *SD *= standard deviation; UK = United Kingdom; US = United States.

^a^*p*-values are from *t*-test.

^b^imputed score.

The AAQoL total score was also comparable between groups (EUR mean = 47.6, NE mean = 48.4, not significant). The AAQoL total score had a 0.8-point difference between groups (EUR mean = 47.6, NE mean = 48.4); the AAQoL total score and section score can range from 0 to 100. Psychological Health (EUR mean = 51.4, NE mean = 52.5, not significant) and Relationships (EUR mean = 53.6, NE mean = 53.7, not significant) section scores were not significantly different between the EUR and NE groups. The differences between EUR and NE groups on the Life Outlook and Life Productivity section scores were significantly different (*p*≤.0004), with the NE group having a higher score on Life Outlook (EUR mean = 45.1, NE mean = 52.5) and the EUR group having a higher score on Life Productivity, (EUR mean = 44.2, NE = 41.2). Life Outlook and Life Productivity section scores were not clinically meaningfully different using standardized differences (standardized differences = -0.440 and 0.161, respectively).

The EQ-5D UK population-based index score and US population-based index score were statistically significantly different (*p*≤.0003), but the differences were very small between groups and not clinically meaningful when calculated with standardized differences (EUR UK mean = 0.80, NE UK mean = 0.84, standardized difference = -0.200; EUR US mean = 0.84, NE US mean = 0.86, standardized difference = -0.143) [28]. The Health State score was significantly lower in the EUR group (70.0) compared with the NE group (75.6, *p*<.0001). When the standardized difference was examined (-0.253), the differences between groups on the EQ-5D UK and US population-based index scores were not clinically meaningful.

There were some statistically significant differences between the EUR and NE groups in education (highest grade completed) (Table 3). As compared with the EUR group, the NE group had a higher percentage of patients achieving college (18.5% vs. 6.6%), post-graduate (6.9% vs. 1.2%), and some college (31.4% vs. 4.4%) education. However, the EUR group as compared with the NE group had a higher percentage of patients reporting secondary education (24.8% vs. 2.8%), vocational training (9.9% vs. 4.0%), and university (13.1% vs. 10.9%) as their highest grade completed.

**Table 3:** Highest grade completed

**Variable**	**EUR group ( *N *=1217)**	**NE group ( *N *=800)**	**Total ( *N *=2017)**	***p*-value**
Highest Grade Completed, *n *(%)				
Number of Patients	1213	799	2012	<.0001^a^
No Formal Education	3 (0.2)	0 (0.0)	3 (0.1)	
Kindergarten	1 (0.1)	0 (0.0)	1 (0.0)	
1st	5 (0.4)	0 (0.0)	5 (0.2)	
2nd	26 (2.1)	1 (0.1)	27 (1.3)	
3rd	5 (0.4)	0 (0.0)	5 (0.2)	
4th	4 (0.3)	0 (0.0)	4 (0.2)	
5th	2 (0.2)	0 (0.0)	2 (0.1)	
6th	4 (0.3)	0 (0.0)	4 (0.2)	
7th	6 (0.5)	0 (0.0)	6 (0.3)	
8th	12 (1.0)	0 (0.0)	12 (0.6)	
9th	31 (2.5)	4 (0.5)	35 (1.7)	
10th	16 (1.3)	10 (1.3)	26 (1.3)	
11th	10 (0.8)	24 (3.0)	34 (1.7)	
12th	49 (4.0)	12 (1.5)	61 (3.0)	
High School	171 (14.1)	130 (16.3)	301 (14.9)	
Primary Education	77 (6.3)	2 (0.3)	79 (3.9)	
Secondary Education	302 (24.8)	22 (2.8)	324 (16.1)	
Vocational Training	121 (9.9)	32 (4.0)	153 (7.6)	
Some College	53 (4.4)	251 (31.4)	304 (15.1)	
College	80 (6.6)	148 (18.5)	228 (11.3)	
University Education	159 (13.1)	87 (10.9)	246 (12.2)	
Some Graduate School	62 (5.1)	21 (2.6)	83 (4.1)	
Post-Graduate	14 (1.2)	55 (6.9)	69 (3.4)	

Abbreviations: EUR = European; *N *= total number of patients; NE = non-European.

^a^*p*-values are from chi-square test.

To determine if education level was the cause of the difference in prior stimulant use between EUR and NE, education level (all levels) was compared with respect to prior exposure to psychostimulants. Overall, prior stimulant use was not related to education level (*p*=.411). Only some college and university education were significantly related to prior stimulant use. Patients who reported their highest education level as some college were more likely to report prior stimulant use (17.6% prior stimulant use vs. 13.7% no prior stimulant use, *p*=.019). Prior stimulant use was reported in fewer patients with university education compared to no prior stimulant use (9.7% prior stimulant use vs. 13.5% no prior stimulant use, *p*=.013).

We further examined whether the difference in level of education as opposed to region was responsible for the differences between groups in AAQoL scores. The Life Outlook score and Life Productivity score were significantly different among education levels (*p*≤.001); however, both scores were affected by a few outliers. There was no substantial difference across education levels in any AAQoL measure.

The summary of present and lifetime diagnosis of comorbid diseases at baseline is shown in Table 4. The current and lifetime diagnoses of most comorbid diseases at baseline were similar between the EUR and NE groups. There was a significant difference between the EUR and NE groups in lifetime diagnosis of depressive disorder (9.0% vs. 4.6%, respectively; *p*<.001) and generalized anxiety disorder (1.8% vs. 0.6%, respectively; *p*=.028), and current diagnosis of depressive disorder (0.6% vs. 0.0%, respectively; *p*=.047), dysthymic disorder (1.7% vs. 0.0%, respectively; *p*<.001), and social phobia (0.6% vs. 0.0%, respectively; *p*=.047).

**Table 4:** Summary of current and lifetime diagnosis of comorbid diseases at baseline

**Psychiatric diagnosis**	**EUR group**	**NE group**	**Total**	***p*-value**^**a**^
	**( *N *=1217)**	**( *N *=800)**	**( *N *=2017)**	
	***n *(%)**	***n *(%)**	***n *(%)**	
Alcohol Abuse				
Current	2 (0.2)	0 (0.0)	2 (0.1)	.521
Lifetime	56 (4.6)	24 (3.0)	80 (4.0)	.080
Depressive Disorder, Not Otherwise Specified				
Current	7 (0.6)	0 (0.0)	7 (0.3)	.047
Lifetime	110 (9.0)	37 (4.6)	147 (7.3)	<.001
Dysthymic Disorder^b^				
Current	21 (1.7)	0 (0.0)	21 (1.0)	<.001
Generalized Anxiety Disorder				
Current	4 (0.3)	0 (0.0)	4 (0.2)	.157
Lifetime	22 (1.8)	5 (0.6)	27 (1.3)	.028
Obsessive-Compulsive Disorder				
Current	2 (0.2)	0 (0.0)	2 (0.1)	.521
Lifetime	10 (0.8)	1 (0.1)	11 (0.5)	.059
Post-traumatic Stress Disorder				
Current	4 (0.3)	0 (0.0)	4 (0.2)	.157
Lifetime	8 (0.7)	3 (0.4)	11 (0.5)	.542
Social Phobia				
Current	7 (0.6)	0 (0.0)	7 (0.3)	.047
Lifetime	16 (1.3)	4 (0.5)	20 (1.0)	.105

Abbreviation: EUR = European; *N *= total number of patients; *n *= number of patients in the specified category; NE = non-European.

^a^*p*-values are from Fisher's exact test.

^b^Lifetime Dysthymic Disorder diagnoses were not collected.

## Discussion

This analysis of adults with ADHD was the first randomized clinical trial of this size analyzing patient characteristics across multiple countries. Results of these analyses of adults with ADHD in the EUR and NE regions indicate that the characteristics of adult patients with ADHD are comparable between the regions. There were some subtle differences, but further evaluation indicates consistency between the EUR and NE groups. The large sample size (*N*=2017) made finding statistically significant differences more likely, but many of these were small in absolute magnitude and were not clinically meaningful.

The familiality of the ADHD diagnosis highlights the need for clinicians to screen parents of children with ADHD [29].

Overall, the CAARS-Inv:SV Hyperactivity-Impulsivity subscale scores and total ADHD symptom score were somewhat higher than those previously seen in US adult ADHD studies, though this difference was not considered clinically meaningful. The CGI-ADHD-S score was slightly lower in the NE group (4.7), but comparable with scores observed in previous US adult ADHD studies [30-32]. The difference between groups was statistically significant, but moderate.

The AAQoL Life Outlook and Life Productivity section scores were statistically significantly different, but arguably of questionable clinical significance. The EQ-5D UK population-based index score, US population-based index score, and Health State score were statistically significantly different, but also arguably of questionable clinical significance.

The NE group primarily included North American countries. Since it also included a small number of patients from Russia and Argentina, a sensitivity analysis was performed to test whether removal of Russian (n=6) and Argentinian (n=27) patient data from the NE data set would change the conclusions of the manuscript; results from this analysis confirmed that, if data from these 33 patients were removed from the NE data set, none of the conclusions would change. Therefore, we kept Russia and Argentina in the NE group to be complete.

The major difference between the EUR and NE groups was prior exposure to psychostimulants. This difference may be due in part to differences in treatment in the EUR and NE regions. In some parts of Europe the use of stimulants to treat adults with ADHD has been controversial and stimulants are not approved for use in adults with ADHD [16]. The 2008 National Institute for Health and Clinical Excellence guidelines in the UK provided guidance for the development of clinical services for adults with ADHD [33]. In fact, previous studies, which looked at prior exposure to psychostimulants in NE ADHD patients, showed higher percentages of patients with prior exposure (43.0% to 46.3% in adults 26 to 77 years of age, 53.8% to 72.4% in young adults 18 to 25 years of age) compared with the current study [31].

Current and lifetime diagnoses of comorbid diseases at baseline were comparable. This may be due in part to the exclusion criteria, which stated that patients should be excluded from the study if they met the criteria for several comorbid conditions. Childhood and adulthood diagnoses of ADHD were also comparable across regions. This could be due in part to the inclusion criteria stating that, to be eligible for the study, patients must meet DSM-IV-TR criteria for current ADHD and for a historical diagnosis of ADHD during childhood, as assessed by the CAADID.

The findings of these analyses on baseline characteristics of the adult ADHD patient in the EUR and NE regions were similar to the conclusion of the previous analyses on the characteristics of children and adolescents with ADHD outside North America [34,35]. In Preuss et al. [35] children across the 10 EUR countries showed similar ADHD symptom severity, coexisting problems, burden of illness, and quality of life, though there appeared to be differences between countries in the use of ADHD diagnostic criteria and treatment. In Buitelaar et al. [34] the effects of atomoxetine on ADHD in non-North American children and adolescents were consistent with North American studies suggesting that results of North American studies in children and adolescents were comparable with patients in other countries. Results of the current study support the validity of the diagnosis of ADHD in EUR adults. The similar ADHD-related characteristics in this study suggest that the findings of North American studies on adult ADHD may be generalized to patients in the EUR region. Improving recognition and treatment standards for adults with ADHD within Europe is important, as untreated ADHD patients have been shown to have higher rates of academic failure, lower occupational status, increased risk of substance abuse disorders, higher rates of accidents, higher rates of marital instability, and fewer social relationships and friends [16,20,36].

Several limitations of our study must be acknowledged. This is a clinical trial patient population that is based on inclusion and exclusion criteria. Due to the homogeneity of the sampling method, the data may not be representative of patients with comorbidities. Though, community-based studies on the prevalence of adult ADHD as part of the World Health Organization World Mental Health Survey found fairly similar prevalence rates of adult ADHD throughout the countries that were surveyed (mean = 3.4% range = 1.2% to 7.3%) [10]. There is also a selection bias, with the population that participates in clinical trials having a greater representation. The study was not prospectively powered to examine differences in ADHD-related characteristics between adult patients in EUR and NE groups. Due to a large sample size (*N*=2017), some comparisons between groups were statistically significant despite the absolute differences being small and not clinically meaningful; hence, *p*-values were used as references only, and interpretation of data was based on the magnitude of the findings rather than the *p*-value. Finally, this study did not look at treatment effects. Despite the limitations of this study, this analysis serves to help define/clarify who the adult ADHD patient is not only in the US, but across several countries.

## Conclusion

In summary, the results reported here show that the characteristics of adult ADHD patients are generally comparable across EUR and NE regions. Although studies in the EUR population may be needed to address specific issues, the results suggest that research findings in adults with ADHD in NE regions can be generalized to adult patients with ADHD in the EUR region.

## Competing interests

Doctors Upadhyaya, Tanaka, Escobar, and Allen are full-time employees of Eli Lilly and Company and stockholders of Eli Lilly and Company. Dr. Arsenault and Mr. Williams are full-time employees of PharmaNet/i3, an inVentiv Health Company. Dr. Arsenault is also a former employee of, and minor stockholder in, Eli Lilly and Company.

Dr. Adler has received royalty payments (as an inventor) from New York University. He receives/d grant research support from Abbott Laboratories, Bristol-Myers Squibb, Chelsea Therapeutics, Merck & Co., Inc., Novartis Pharmaceuticals Corporation, Shire, Eli Lilly and Company, Ortho-McNeil/Janssen/Johnson & Johnson, New River Pharmaceuticals, Cephalon Inc, Organon, and National Institute of Drug Abuse. He is/has been on the advisory board and consulted for Alcobra Pharmaceuticals, AstraZeneca, Shire, Eli Lilly and Company, Mindsite, Major League Baseball, and i3 Research. He is/has been a consultant for Epi-Q, INC Research, United Biosource, Otsuka, Major League Baseball Players Association, Ortho-McNeil/Janssen/Johnson & Johnson, Psychogenics, and Theravance.

Dr. Casas has received travel grants from Eli Lilly and Company, Janssen Cilag, Shire, and Laboratorios Rubió. He receives/d grant research support from Janssen Cilag, Shire, Laboratorios Rubió, and Eli Lilly and Company. He is/has been on the advisory board for Janssen Cilag, Shire, Laboratorios Rubió, and Eli Lilly and Company. He is/has been a consultant for Janssen Cilag, Shire, Laboratorios Rubió, and Eli Lilly and Company.

Dr Kutzelnigg has received travel grants from Eli Lilly and Company, Affiris AG, Novartis Pharmaceuticals Corporation, and AstraZeneca and payment for lectures including service on speakers’ bureaus from Eli Lilly and Company, Novartis Pharmaceuticals Corporation, and Affiris AG, and serves as a consultant for Eli Lilly and Company.

## Authors' contributions

HU made substantial contributions to the analysis and interpretation of the data, was involved in drafting the manuscript and revising the intellectual content of this manuscript. LAA made substantial contributions to the analysis and interpretation of the data, was involved in drafting the manuscript and revising the intellectual content of this manuscript. MC was involved in drafting the manuscript and revising the intellectual content of this manuscript. AK made substantial contributions to the analysis and interpretation of the data, was involved in drafting the manuscript and revising the intellectual content of this manuscript. YT made substantial contributions to the design of the study, performed the statistical analysis, and was involved in drafting the manuscript and revising the intellectual content of this manuscript. DW performed the statistical analysis, and was involved in drafting the manuscript and revising the intellectual content of this manuscript. JA was involved in drafting the manuscript and revising the intellectual content of this manuscript. RE made substantial contributions to the design of the study, analysis and interpretation of the data, and was involved in drafting the manuscript and revising the intellectual content of this manuscript. AJA made substantial contributions to the design of the study, analysis and interpretation of the data, and was involved in drafting the manuscript and revising the intellectual content of this manuscript. All authors read and approved the final manuscript.
